# Genetic Parameters of Linear Type Traits Scored at 30 Months in Italian Heavy Draught Horse

**DOI:** 10.3390/ani10061099

**Published:** 2020-06-25

**Authors:** Fabio Folla, Cristina Sartori, Enrico Mancin, Giuseppe Pigozzi, Roberto Mantovani

**Affiliations:** 1 Department of Agronomy, Food, Natural Resources, Animals and Environment—DAFNAE, University of Padova, Viale dell’Università 16, 35020 Legnaro (PD), Italy; max_fabio@libero.it (F.F.); enrico.mancin@phd.unipd.it (E.M.); roberto.mantovani@unipd.it (R.M.); 2 Italian Heavy Draught Horse Breeders Association (ANACAITPR), 37068 Vigasio (VR), Italy; direzione@anacaitpr.it

**Keywords:** horse, linear type traits, genetic evaluation, selection, Italian Heavy Draught Horse

## Abstract

**Simple Summary:**

Current selection in the Italian Heavy Draught Horse (IHDH) is based on linear type traits scored on foals, but the studbook admission of candidate mares and stallion requires an additional evaluation of linear type traits at adult age, about 30 months. The study intended estimating the genetic parameters of these traits to evaluate the possible shift from type scored on foals to adult. Results showed a moderate heritability of traits, and a positive genetic trend was observed in the traits of selection interest. This suggests the feasibility of using linear type traits scored at adult age for genetic improvement of the IHDH.

**Abstract:**

The Italian Heavy Draught Horse (IHDH) breed is selected based on linear type traits (LTT) evaluated at young age on six-month-old foals. However, animals retained for reproduction are scored also at adults age (about 30 months), and the evaluation is mandatory for the final official admission to the stud book of candidate mares and stallions. This study aimed to estimate genetic parameters of LTT scored at 30 months to consider if they are feasible for selection instead of using foal data and to reduce costs of selection plan. Data included 19 years of evaluation for 14 LTT and an overall score. Analyses were performed on 5835 females and 856 males via animal model. The heritability ranged from 0.03 (upper line length) to 0.40 (frame size). Traits of selection interest (head size and expression; temperament/movement; fleshiness; fore diameter; rear diameter) reported heritability between 0.21 and 0.31. High genetic correlations were obtained among traits related to muscular development, 0.73 on average. Positive genetic trends were found in traits of selection interest, already selected from foal type trait data. Accounting for genetic parameters estimated in adult animals instead in foals is feasible in IHDH selection.

## 1. Introduction

The goal of breeding organizations and breeders is to improve the genetic value of animals over generations. Breeding for conformation accounts aspects as the morphology and movement, and it is an important aspect of breeding decisions in many livestock species including pigs, sheep, cattle and horse [1]. Traits may be measured or scored (in points), depending on the breeding goals. If scored, evaluators provide a subjective judgement of the correctness of the trait [2]. In some horse breeds a linear evaluation system developed in dairy cattle [3] was introduced in the 1990s (e.g., in Dutch Warmblood [4]; in Italian Haflinger [5]). Under this system, extreme scores correspond to the biological extremes of the trait, and individual scores lie between these extremes. Heritabilities of linear traits are consistent with the ones obtained through a subjective scoring, as reviewed for various breeds [2]. The current conformation of a horse is the result of both natural and breeders’ selections, and the traits evaluated for each breed depend on the breeding purposes. While head, neck and shoulders are evaluated almost in all horse breeds, horses bred for racing and riding performances are also scored for the regularity of gaits, the walk and the trot, whereas evaluation in draught horses put great attention on fore and rear quarters [2,6]. Horses used for meat production are also evaluated for traits related muscle development, such as diameter or fleshiness in different body areas (e.g., [7,8]). This latter trait is typically considered in beef cattle, while for horses it is only evaluated in the Italian Heavy Draught Horse (IHDH) [9,10]. The achievement of the conformation standards typical of the breed is a first requirement for the admission to the stud book. In the breeding program of e.g., the Royal Dutch Warmblood, two different types of traits are recorded: descriptive (as walk and trot, scored linearly) and subjective (overall conformation and movement, valued from very bad to excellent). Evaluation is usually done at 3–7 years of age, and involves most of the young horses of the breed [11]. Regarding draught breeds, evaluation of e.g., the Noriker horse occurs at three years or older and currently involves linear traits [12], whereas past evaluation regarded body measurements [6]. Posavje horses are evaluated at 30–60 months, using both body measurements and linear scores [13]. Various methods of evaluation are used in different breeds (e.g., linear traits in the Pura Raza Español horse, also known as Andalusian [14]), most of which are evaluated once in life. Some examples of morphologic evaluations in horses are reported in Table 1. Recently, some pilot studies of image analysis (digital measures) have been done to evaluate morphologic traits. An overview of linear scores modeling in warmblood horses was done by Duensing and colleagues [1].

The IHDH (Supplementary Figure S1) is a native horse breed originated in middle of the 19th century by the Italian government. It originates mainly from crosses of Norfolk–Breton stallions from France with local mares of the northeast of Italy to obtain a heavy strain of horse to be used in both agriculture (heavy draught) and field artillery. Nowadays the breed is still used for heavy draught in agriculture, as in the past, but also for leisure activities, and mainly for meat production [43]. The last official update of the FAO breeds database (August 2019; fao.org/dad-is) reported a population size for IHDH of 5137 individuals, including 353 stallions and 2962 mares. Selection in IHDH is based on the genetic evaluation for linear type traits since 1992 [43]. Genetic improvement in this breed has the dual purpose of meat and draught: the main selection goal is meat production, involving about 85% of the young male foals and less than 20% of the females, with typical slaughter ages at about 12 or 18 months [44]. However, in recent years, an increasing interest for the original heavy draught attitude has occurred, and it has implied the use of horse in team races and in agricultural works in the organic farms [43,45]. Genetic evaluation is based on a linear scale scoring system and is performed two times in life: a first evaluation when animals are foals, at about six months and a latter evaluation at 30 months of age. Since horses reach almost the complete somatic development at an age of 24 months [46], animals at 30 months can be already considered as young mares and young stallions (later called just “young mares and stallions”). The linear type evaluation at six months involves the scoring of 11 traits, that can be assigned to the three main groups of general aspect, trunk and legs [9,43]. The linear evaluation at 30 months uses the same scale used at a six-month evaluation. Moreover, three traits about the correctness of feet and legs are added, as well as an additional overall score of conformation [10].

Genetic improvement is based just on the linear type traits obtained on six-month-old foals, five of them weighted in a total merit index (TMI) indicating the selective value of the horse [9,47]. The traits involved in total merit index and the respective economic weights within TMI (in brackets) are head size (0.25), temperament/movement (0.15), fleshiness (0.25), fore diameter (0.15), rear diameter (0.20). Before to obtain the genetic evaluation a preliminary admission to the foals register of the studbook is possible for males if they have at least three generations of known ancestors, a minimum pedigree index (obtained as average of EBVs of parents) for TMI of 100 (the index is set with mean at 100 and standard deviation at 10 [43]) and a minimum final morphologic score obtained at six months of “good” (subjective scoring in six points from “unfair” to “excellent”). On the other hand, for females, three generation of known ancestors and a final morphologic score of “fair” are sufficient. If these requirements are satisfied, male and female foals are linearly scored at six months, the genetic evaluation of linear type traits is performed and individual TMI are calculated and used for breeding purposes.

Traits scored on 30-month animals are not directly used for genetic improvement, but they are mandatory for the final admission of candidate stallions and mares to the stud book [10]. They are not used indeed for estimating genetic parameters, but only as a phenotypic score to be joined to the TMI calculated on linear type traits of foals. The estimation of genetic parameters in linear type traits scored at 30 months has been not performed yet, although the use of traits scored at this age, instead of at six months, could be interesting for the IHDH genetic improvement. Indeed, evaluating animals only at 30 months could lead to a reduction of the costs of the whole selection process (about 450 young mares and stallions/year are evaluated vs. 800 foals/year). This saving could be useful due to the occurring shortage of funding to breeders associations [10]. The advantage of using traits scored on six-month foals is to speed up genetic progress for selected traits due to the young age of animals used, but a sound genetic improvement could occur also using 30-month scoring. Furthermore, 30 months is closer to the age at which most of horse breeds are evaluated for the admission to stud book (e.g., in Andalusian horse; [14]; see also Table 1).

Following these considerations, this study aimed to estimate genetic parameters of liner type traits in the IHDH evaluated at the age of about 30 months, when animals are young mares and stallions. Moreover, the study also aimed to assess the genetic correlations among these traits and to estimate the genetic trends realized for traits. In terms of genetic improvement of the breed, this is a challenge of using linear traits scored at 30 months rather than obtained at six months.

## 2. Materials and Methods

### 2.1. Description of Data

An amount of 7133 records was obtained from data of the stud book of IHDH breed and from the linear type traits information routinely recorded by the National breeder association (ANACAITPR; anacaitpr.it). Records prior to 1992, as well as horses without father and mother, horses lacking stud-farm, birth date or evaluation date were eliminated.

The dataset suitable for analyses included single records of 6691 horses (5835 females, 856 males), aged about 30 months (the 95% of animals is aged between 26.95 and 68.30 months, with a median of 30.19 months) and 11,012 individuals in pedigree. Linear type traits scored by 33 classifiers in 19 subsequent years of evaluation were considered. As in foals, linear type evaluation used a 9-point scale system (from 1 to 5, including half points).

The traits were classified in three classes as follows: (i) Traits of general aspect: head size and expression (HS), temperament/movement (Te/M), frame size (FS), fleshiness (Fl), bone incidence (BI); (ii) Traits of the trunk: thorax depth (TD), fore diameter (FD), rear diameter (RD), upper line length (UL), upper line direction (UD); (iii) Traits of the limbs: legs side view (LS), fore feet (FF), rear feet (RF), hind legs back view (HL). In IHDH breeding management, animals with a light head (HS) are preferred for selection, as well as with a great reactivity to environmental stimuli and a regular trout (Te/m). High scores of FS must be preferred for selection. A great development of muscles masses of croup, thigh, buttock, loins and withers (all considered for scoring Fl), a fine-boned frame (BI), a depth thorax (TD) and large chest (FD) and croup (RD) are also desirable. Intermediate optima are related to traits scoring the correctness of conformation that are UL, UD, LS, FF, RF and HL. The last three traits are scored only at 30 months. A detailed description of the traits is reported in Supplementary Table S1.

The study also considered the overall score (OS) of conformation, subjectively scored only on 30-month animals and assigning a final morphologic judgement from “fair” to “excellent”.

The traits measured at six months were not included in the present study, just focused on traits of 30-month animals. Anyway, an evaluation of the genetic correlation among six and 30 month-traits is currently under study and could be useful for future decisions about linear type traits evaluations.

### 2.2. Estimates of (Co)Variance Components, Correlations and Genetic Trend

The non-genetic effects considered for the analysis included the sex of the animals and the age at scoring (in month). The combined effect of the classifier and of the year of evaluation was also considered, as for Italian the Haflinger [5].

A variable called “stud group” was formed to consider the effect of the small studs, i.e., with less than two animals scored within a year of evaluation. This was carried out on the basis of geographical position and management (stable, pasture and stable or outdoor), the farm’s production goal (production of foals for heavy draught or meat), the general prophylaxis on foals (vaccination or not) and the mean value mares ‘body condition registered at foals’ evaluation. In this way, groups were created for neighboring studs with similar nutrition and management. A detail description of stud group constitution and evaluation has been reported in a previous study on linear type traits in IHDH foals [9]. Therefore, the effect included in the model was a combination between the group of studs (stud group), the year of birth of foal and the classifier.

A preliminary ANOVA (PROC GLM; SAS Inst. Inc., Cary, NC, USA) was run on the non-genetic effects to be included in the genetic model: the combination of stud-group classifier—year of birth (SYC, 1663 levels); the sex of animals (2 levels); the age at scoring (5 classes, i.e., ≤27, 28, 29–32, 33–47 and ≥48 months of age). The classes of age at scoring were built on the basis of the frequency of the individual ages.

Variances of traits and (co)variance components for all the 14 linear type traits and the OS and for each pairwise combination between traits, were estimates via Average Information REML method (AIREML [48]) using single-trait and bi-trait animal models and running the AIREMLF90 program, part of the BLUPF90 software suite (Athens, GA, USA) [49]. Preliminary AIREML analyses included the SYC effect either as fixed or random. Looking at the Akaike Information Criterion (AIC [50]) as model fitting statistics obtained running the analysis, the final model included this effect as fixed and was written as follows:

**y** = **Xβ** + **Zu** + **e** (1)

where: **y** was the vector of observations for one of the 14 traits recorded on a single animal; **β** was the vector of the same fixed effects of the ANOVA; **u** was the vector of the random additive genetic effect (11,012 levels, as the animals in pedigree); **e** was the vector of the random residual terms; **X** and **Z** were the incidence matrices assigning observations to the related effects.

The assumptions about the structure of (co)variances for bivariate analysis run on each trait pair were written as:

(2) $$Var\left|\begin{array}{c} u\\ e \end{array}\right|=\left|\begin{array}{cc} G⊗A & 0\\ 0 & R⊗I \end{array}\right|;G=\left|\begin{array}{cc} \sigma_{a1}^{2} & \sigma_{a12}\\ \sigma_{12} & \sigma_{a2}^{2} \end{array}\right|;R=\left|\begin{array}{cc} \sigma_{e1}^{2} & \sigma_{e12}\\ \sigma_{e12} & \sigma_{e2}^{2} \end{array}\right|$$

where G was an additive genetic covariance matrix of order 2 × 2, A the additive genetic relationships matrix for p animals, R a residual covariance matrix of order 2 × 2, I an identity matrix, ⊗ is the Kronecker product operator, $\sigma_{a1}^{2}$, $\sigma_{a12}$, $\sigma_{a2}^{2}$ are, respectively the additive genetic variances for the two traits and their covariance and $\sigma_{e1}^{2}$, $\sigma_{e12}$, $\sigma_{e2}^{2}$ the residual (co)variances for the traits.

The standard errors of the heritability ($SE_{h^{2}}$) were computed following Lynch and Walsh [51]:

(3) $$SE_{h^{2}}=h^{2}\times {\left(\frac{Var\left(\sigma_{a}^{2}\right)}{{\left(\sigma_{a}^{2}\right)}^{2}}+\frac{Var\left(\sigma_{p}^{2}\right)}{{\left(\sigma_{p}^{2}\right)}^{2}}-\frac{2Cov\left(\sigma_{a}^{2},\;\sigma_{p}^{2}\right)}{\sigma_{a}^{2}\sigma_{p}^{2}}\right)}^{0.5}$$

where $h^{2}$ is heritability of a given trait, $\sigma_{a}^{2}$ and $\sigma_{p}^{2}$ are the additive genetic and phenotypic variances of the trait, $Var\left(\sigma_{a}^{2}\right)$, $Var\left(\sigma_{p}^{2}\right)$ are their respective predicted error variances and $Cov\left(\sigma_{a}^{2},\;\sigma_{p}^{2}\right)$ is the predicted error (co)variance. Furthermore, standard errors of genetic and phenotypic correlations (SE_r_) were computed as follows [51]:

(4) $$SE_{r}=r\times {\left(\frac{Var\left(\sigma_{1}^{2}\right)}{4{\left(\sigma_{1}^{2}\right)}^{2}}+\frac{Var\left(\sigma_{2}^{2}\right)}{4{\left(\sigma_{2}^{2}\right)}^{2}}+\frac{Var\left(\sigma_{12}\right)}{\sigma_{12}^{2}}+\frac{2Cov\left(\sigma_{1}^{2},\;\sigma_{2}^{2}\right)}{4\sigma_{1}^{2}\sigma_{2}^{2}}-\frac{2Cov\left(\sigma_{1}^{2},\;\sigma_{12}\right)}{2\sigma_{1}^{2}\sigma_{12}}-\frac{2Cov\left(\sigma_{12},\;\sigma_{2}^{2}\right)}{2\sigma_{12}\sigma_{2}^{2}}\right)}^{0.5}$$

where r is the correlation between the two traits (genetic, *r_g_* or phenotypic, *r_p_*) and the other terms are the (co)variances of the traits (1 and 2) and their predicted error (co)variances. Significance of phenotypic and genetic correlations was tested following Kohn and Atchley [52].

The genetic trends for all traits of the study were traced from the average breeding values (EBVs) of individuals born in the same year found running a BLUP analysis after AIREML estimations (BLUPF90 program of BLUPF90 software suite (Athens, GA, USA) [49]). EBVs were standardized to have mean value of 100 and standard deviation of 10.

## 3. Results

### 3.1. Description of Data

Means and standard deviations of traits, as well as minimum and maximum values of their scores are reported in Table 2. The means ranged from 2.05 (overall score) to 3.56 (thorax depth) and standard deviations were in the range from 0.33 (hind legs back view) to 0.80 (overall score). Most of traits had a mean close to 3, that is the mean point of the linear scale. The traits showing the higher mean evaluations were thorax depth (3.56), rear diameter (3.38), upper line direction (3.30), temperament/movement (3.29) and fleshiness (3.29). Conversely, the traits exhibiting the lowest evaluations were fore feet (2.05), legs side view (2.55), bone incidence (2.86) and upper line direction (2.83). Higher values are preferred for all traits apart the ones with intermediate optima, which desirable value is 3. However, rear feet are the only trait with a mean value that is almost 3. Standard deviations of traits ranged from 0.33 (hind legs back view) to 0.79 (overall score).

Table 2 also reported skewness and kurtosis values. Skewness ranged from −1.80 (upper line direction) to 0.65 (upper line length). A strong right asymmetry of the distribution (negative values of skew) was also found for bone incidence and hind legs back view, whereas a moderate left asymmetry (positive values of skew) was reported for fore feet and overall score. Fleshiness, rear diameter and rear feet showed range values near zero. Kurtosis values ranged from −1.06 (legs side view) to 4.40 (hind legs back view). Most of traits showed a moderately broad distribution (negative values of kurtosis), whereas bone incidence and the traits with intermediate optima excluding LS reported a narrow distribution.

### 3.2. Estimates of (Co)Variance Components

The results of preliminary ANOVA are reported in Supplementary Table S2. The SYC effect was significant for all traits considered (*p* < 0.001), as well the sex, except for temperament, thorax depth, rear diameter, legs side view, hind legs back view and overall score. The age at evaluation was significant only for temperament, frame size, fleshiness, thorax depth, fore and rear diameter, legs side view and overall score. Residual variance, expressed as root mean square error, ranged from 0.08 (hind legs back view) to 0.43 (overall score).

Estimated variances, heritability and standard error are presented in Table 3. For all traits under study, the AIC value (data not shown) obtained running the preliminary analyses resulted lower when SYC effect was considered as fixed, allowing to include this effect as fixed in the final analyses.

The traits showing the greatest genetic variance (Table 3) were frame size (σ_a_^2^ = 13.98), overall score (σ_a_^2^ = 13.47) and head size (σ_a_^2^ = 10.09), while the traits with the lowest values were upper line direction (σ_a_^2^ = 0.28), rear feet (σ_a_^2^ = 0.40) and hind legs back view (σ_a_^2^ = 0.54). The standard errors (SE) of genetic variances ranged from 0.19 (legs back view) to 1.47 (overall score). Residual variances were higher than genetic ones; higher values were found for overall score (σ_e_^2^ = 30.22), head size (σ_e_^2^ = 22.20) and frame size (σ_e_^2^ = 20.92), whereas lower values were found for legs back view (σ_e_^2^ = 7.98) and upper line direction (σ_e_^2^ = 8.41). Their SE values were in the same range of the ones of the genetic variances.

Heritabilities obtained for linear type traits were low or moderate depending on traits and ranged from h^2^ = 0.03 to h^2^ = 0.40. Looking at the results, the most heritable traits were frame size (h^2^ = 0.40), head size (h^2^ = 0.31), fore diameter (h^2^ = 0.31) and overall score (h^2^ = 0.31) while the lowest values were obtained for the correctness traits of upper line direction (h^2^ = 0.03), rear feet (h^2^ = 0.03), legs back view (h^2^ = 0.06) and fore feet (h^2^ = 0.08). Temperament/movement, fleshiness, thorax depth and rear diameter had moderate heritabilities (ranging from h^2^ = 0.21 to h^2^ = 0.27). Standard errors of heritability were low and ranged from SE_h_^2^ = 0.017 (rear feet) to SE_h_^2^ = 0.033 (frame size).

### 3.3. Genetic and Phenotypic Correlations

Estimates of genetic and phenotypic correlations between traits pairs are reported in Table 4. An extended version of table including the approximate standard error of traits is reported as supplementary material (Supplementary Table S3). The most negative genetic correlation (*r_g_*) were found between upper line direction and rear feet (*r_g_* = −0.99) and between bone incidence with both temperament (*r_g_* = −0.74) and head size (*r_g_* = −0.64). Legs side view was negative correlated with most traits (*r_g_* from −0.39 to 0.25, but some *r_g_* did not differ from zero). The greatest *r_g_* were found between fleshiness and both fore diameter (*r_g_* = 0.74) and rear diameter (*r_g_* = 0.91) and between rear diameter with fore diameter (*r_g_* = 0.85). Thorax depth showed as well a great *r_g_* with fleshiness (*r_g_* = 0.55), fore and rear diameter (*r_g_* = 0.56, *r_g_* = 0.74). The trait was also highly correlated with frame size (*r_g_* = 0.71), the latter highly correlated also with rear diameter (*r_g_* = 0.73) and moderately with fleshiness (*r_g_* = 0.45), fore diameter (*r_g_* = 0.52) and upper line length (*r_g_* = 0.41) and direction (*r_g_* = 0.38). Moderate and significant positive *r_g_* of bone incidence regarded frame size (*r_g_* = 0.22) and fore feet (*r_g_* = 0.36). This latter trait was also positively related with fleshiness (*r_g_* = 0.33) and fore diameter (*r_g_* = 0.35). Head size has a great positive *r_g_* with temperament/movement (*r_g_* = 0.67) and a moderate, but significant r_g_ with fore diameter (*r_g_* = 0.18). Temperament/movement was moderately correlated also with frame size (*r_g_* = 0.24) and legs back view (*r_g_* = 0.25). The overall score was significantly positively correlated with most of traits: head size (*r_g_* = 0.45), temperament/movement (*r_g_* = 0.47), frame size (*r_g_* = 0.85), fleshiness (*r_g_* = 0.61), thorax depth (*r_g_* = 0.72), fore and rear diameter (*r_g_* = 0.70, *r_g_* = 0.77). Negative, but not different from zero *r_g_* were found only with fleshiness, rear feet and legs back view. The approximate genetic standard errors ranged from SE*r_g_* = 0.007 (udder depth vs. rear size) to SE*r_g_* = 0.327 (udder depth vs. legs side view). Genetic correlations with great SE*r_g_* were almost not different from zero.

Generally phenotypic correlations (*r_p_*) had the same sign, but lower values than the respective genetic correlations. Different signs were found when the correlations were not different from zero. The trait that showed the greatest correlations with the others was the overall score with frame size (*r_p_* = 0.58), rear (*r_p_* = 0.54) and fore diameter (*r_p_* = 0.52). Rear diameter exhibited as well a positive and medium-high correlation with fleshiness (*r_p_* = 0.51), as well as fore diameter (*r_p_* = 0.50). Fleshiness showed positive *r_p_* with fore diameter (*r_p_* = 0.47) and rear diameter (*r_p_* = 0.51). Another positive *r_p_* was fore between diameter with frame size (*r_p_* = 0.47). The legs side view was negatively correlated with most traits (*r_p_* from −0.10 to 0.11). The lowest *r_p_* were found between head size and bone incidence (*r_p_* = −0.29) and temperament/movement with bone incidence (*r_p_* = −0.18). The standard errors of *r_p_* were lower than genetic ones and ranged from SE*r_p_* = 0.011 (overall score vs. temperament/movement) to SE*r_p_* = 0.016 (head size vs. fleshiness).

### 3.4. Genetic Trends of Traits

**Figure 1:**
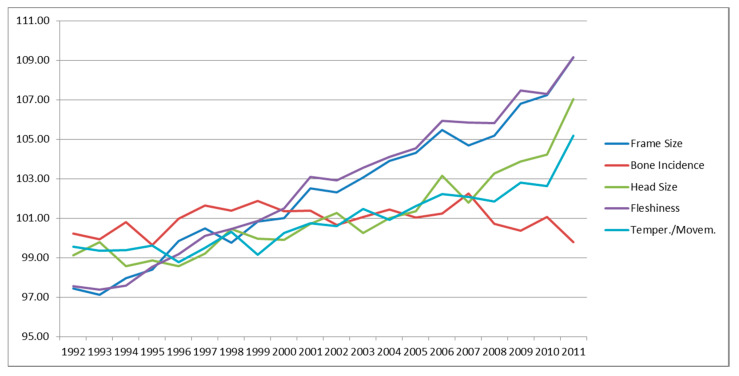
Genetic trends for general linear type traits.

**Figure 2:**
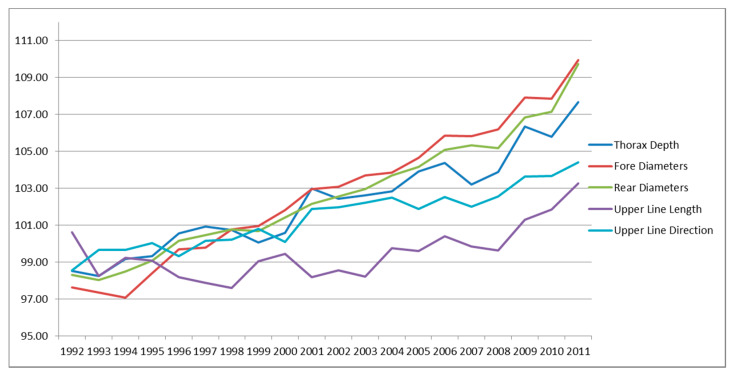
Genetic trends for linear type traits of trunk.

**Figure 3:**
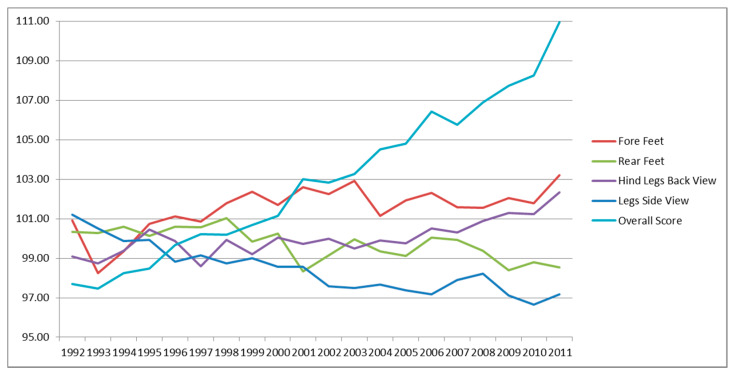
Genetic trends for legs traits and overall score.

Figure 1, Figure 2 and Figure 3 reports the genetic variation of traits over time. Traits recorded in young age are routinely used for the genetic improvement, then looking at the trend of traits recorded at 30 months it is possible see that results of selection. Figure 1 shows the traits of general aspect. Among them, the traits involved in TMI (head size, temperament/movement and fleshiness) had a positive trend. Frame size—that includes traits under selection in its computation (fore diameter and rear diameter)—followed the trend of the other traits. Bone incidence, not included in the TMI, showed a negative trend.

Figure 2 reported the trunk traits, among which the traits with the greatest positive trend were fore and rear diameter, both traits involved in the TMI. Thorax depth showed a positive trend as well, despite not included in TMI. Upper line length and direction, not selected and with intermediate optima, had a positive, but lower trend.

Figure 3 shows the limb traits. None of these were included in the TMI; all of them show an intermediate optima. Fore feet and hind legs back view showed a low genetic increase over the years, while rear feet and legs side view display a low negative trend. The overall score, also reported in Figure 3, showed an increase over time, meaning that the selection carried out in young foals is effective also for 30-month animals.

## 4. Discussion

A first estimation of genetic parameters for type traits recorded in IHDH at the age of 30 months, when animals are young mares and stallions, was provided in the present study. Genetic evaluation for IHDH, based on linear scoring, offers new insights into the framework of the genetic studies about conformation traits in horse breeds. The IHDH, indeed, is currently the only horse breed in which genetic parameters were estimated for traits expressly scored for meat production. Furthermore, the evaluations considered in this study were realized at the age in which in many horse breeds individuals are scored to be admitted at stud book. An overview of the age at scoring in different horse breeds is reported in Table 1. Horses are usually valued at the age of about 3–4 years or more, as reported for saddle horses like the Dutch Warmblood horse [53] and for the Andalusian [14]. An age at evaluation of at least 30 months has regarded also draught breeds such as the Italian Haflinger [17], the Noriker [12], the Bardigiano [17] and the Posavje [13], despite draught horses are generally considered early maturing [6]. The IHDH is currently valued at 2–7 months of age to ease a rapid genetic improvement [43], but some differences in the heritable components of traits may be disclosed in six months [9] and in 30-month-old animals. As examples, in 30-month horses, frame size h^2^ is greater of 0.14, maybe due to a greater genetic variability in growth, whereas fleshiness h^2^ decreases of 0.10, maybe because after selection young mares and stallions could show a reduced variability than foals.

Table 1 also provides an overview of the evaluation systems for conformation that have been applied to horse breeds over the years. These data are difficult to compare, because noteworthy differences in breeding goals and in evaluation systems occur among breeds [6]. The direct measurement of body regions has been widely accounted in horse literature, such as in the Andalusian horse [14], the Lipizzan [26] and the Noriker [6]. Morphometric measurements have received recent improvements by the software for image analysis of individual body pictures, as for the Spanish Arab Horse [38], and, more recently, for the Lipizzan horse [54,55]. Since the first evaluation system proposed in 1989 for the Dutch Warmblood [4], many studies have been based on linear scoring system, in which a number of traits are individually scored along a biological scale to evaluate body regions (e.g., in Shetland pony [37]; in the Italian Haflinger [5]). In some other cases, traits are subjectively scored (e.g., in the Trakehner horse [56]) or are a combination of biologic and subjective scoring (e.g., in Noriker [6]; in the Bardigiano [17]). Linear traits, as well as morphometric measurements, have been widely introduced over the years because they provide more objective methodologies assessing conformation than traditional subjectively scoring. This system, indeed, is typically more influenced by environmental factors [2]. Linear scoring system also allows to score a large number of conformation traits individually rather than in combination [31]. Scoring traits individually may allow to more easily reveal the differences in conformation between animals, than situations in which different traits are combined [6].

Linear type evaluation in IHDH involves 12 traits that are individually scored plus two traits that are a combination of others, frame size and fleshiness. Differently, the final overall score of conformation, considered only at 30 months evaluations, is subjectively scored. Genetic parameters estimated in the present study concern several traits that have been also valued in a number of horse populations, like the traits related to correctness of body and legs. But looking at the traits related to muscular development (summarized in fleshiness in IHDH), the estimates obtained in this study are difficult to compare within other horse breeds, due to the lack of similar studies. The IHDH a bulky horse mainly selected for meat, so comparison can be made with cattle hypertrophic breeds. As a matter of fact, some associations of traits under selection and myostatin gene (MSTN), which functional mutations contribute to hyper-muscularity in various mammal species, have been recently found in IHDH [57]. Looking at literature in beef cattle, heritability estimates reported in Piedmontese young bulls [58] ranged from h^2^ = 0.26 to h^2^ = 0.55. A further analysis on Piedmontese cows [59] reported an average heritability of h^2^ = 0.12 for linear type traits scoring muscles in withers, shoulder, loin and thigh. Again, heritabilities between h^2^ = 0.36 and h^2^ = 0.41 for traits correlated with thigh muscularity were found in Belgian Blue cows [60], while a value of h^2^ = 0.22 was reported for trait correlated with muscularity (thigh development) in Spanish Asturian beef cattle [61]. Then, values ranging from h^2^ = 0.25 to h^2^ = 0.34 for shoulder, back and rump muscling scores were found in Czech Beef Cattle [62]. Another trait important for meat evaluation is bone incidence since it is a reliable indicator of the further incidence of bones in the animal carcass. This trait is not scored in horses excluding IHDH but recorded in beef cattle. An estimation of the heritability of the trait (here called bone thinness) was reported, e.g., for Piedmontese cattle [59] and was close (h^2^ = 0.12) to the one of this study.

Other traits of interest for meat purpose, fore diameter and rear diameter, were valued also in other horse breeds than IHDH because they are important for the heavy draught. Heritability values not in agreement with those obtained in this study were reported by other Authors that linearly scored the same body part. Specifically, Druml et al. [6] found a h^2^ = 0.16 for fore quarter in Noriker horse, whereas Molina et al. [14] and Miserani et al. [33] estimated heritabilities of 0.40 and of 0.51, respectively, for chest width in Andalusian and Pantaneiro horses. One of the first estimations on linear type traits, performed by Van Bergen and Van Arendonk on the Shetland pony [37], reported a h^2^ = 0.18, whereas the work of Vicente et al. [28] on Lusitano horse found a lower value of h^2^ = 0.12 on chest and thorax trait. Finally, Bakhtiari et al. [25] found a value of h^2^ = 0.22 for the morphometric measurement of chest width in the Iranian Thoroughbred. Some works [14,28] included in the same evaluation both chest and thorax, that are separately scored in IHDH evaluation, providing different heritabilities (h^2^ = 0.40 in Andalusian and h^2^ = 0.12 in the Lusitano horse).

The rear diameter heritability found in IHDH in the present study is higher than those found in the Italian Haflinger [5] for croup width (h^2^ = 0.11) and in the Lusitano horse for croup ([28]; h^2^ = 0.15). Values of heritability similar to the present study were found in the Dutch Warmblood ([4]; h^2^ = 0.28), in the Noriker ([6]; h^2^ = 0.20) and in the Bardigiano ([7]; h^2^ = 0.25), whereas a greater value of h^2^ = 0.59 was found in the Pantaneiro horse [33] for croup height.

The combined trait of frame size reported heritability greater than the values of the single traits constituting this phenotype that are diameter, thorax and of the height. An overall evaluation of the frame was also found in some other horse breeds, such as the Hanoverian Warmblood ([23]; h^2^ = 0.20).

Regarding the other traits under selection in IHDH, head size is widely evaluated in horse breeds regardless the breeding purpose. Lower heritabilities than in this study were found in Noriker ([6]; h^2^ = 0.11) and in Italian Haflinger ([5]; h^2^ = 0.24 as average of head volume and expression). Similar heritability values were found for head and neck in Andalusian horses ([14]; h^2^ = 0.23), in Dutch Warmbloods ([4]; h^2^ = 0.21) and in Lusitanos ([28]; h^2^ = 0.18). Differently, in the Bardigiano horse three h^2^ values of head (shape: h^2^ = 0.20^,^ profile: h^2^ = 0.26 and expression: h^2^ = 0.32) were reported [17]. In the Pantaneiro horse, different heritabilities for head length (h^2^ = 0.55) and head width (h^2^ = 0.27) were found [33]. Again, a high heritability of 0.47 was reported for head in the Hanoverian Warmblood [23]. Finally, a value of h^2^ = 0.39 for the morphometric measure of head length was found in the Iranian Thoroughbred [25]. The great differences among h^2^ estimates may be explained by breed variability, the selection goal and the evaluation method (e.g., in the Haflinger, the Andalusian and the Noriker a scale from 1 to 10 is used, while in the Iranian Thoroughbred body measurements are taken). About IHDH, a light head is preferred (higher score) because it relies with a greater elegance of the individual and also correlates with a lower bone incidence, preferred for the meat purpose.

The last trait included in the selection index, the temperament/movement, is not a type traits, but it is often scored in horse breeds because it concurs to the general framework of individual body appearance. In IHDH the trait evaluated both the docility and the regularity of the movement, often separately considered in other horse breeds. The heritability for this trait is similar to the value found in the Bardigiano ([17]; h^2^ = 0.19) for the same trait and in the Haflinger for gait ([5]; h^2^ = 0.19). In this breed a low heritability (h^2^ = 0.06) was found for temperament, but the definition of the trait is a bit different than in IHDH. Furthermore, in Andalusian [14] a low h^2^ (0.08) was found for temperament. Notwithstanding, movement, which definition partly overlaps the one of temperament in IHDH, had an heritability of 0.15 in this Andalusian and of 0.20 in the Noriker [6], that are similar to the heritability of temperament/movement in IHDH.

The heritabilities of the traits scoring the correctness of body and legs are low; this is probably because they are related to a proper conformation of the animal and have intermediate optima. The heritabilities for the linear scores of legs ranged from h^2^ = 0.07 to h^2^ = 0.21 in Shetland pony [37], from h^2^ = 0.14 to h^2^ = 0.23 in Dutch Warmblood horse [4], from h^2^ = 0.10 to h^2^ = 0.17 in Italian Haflinger [5], from h^2^ = 0.05 to h^2^ = 0.24 in the Bardigiano [17] and of h^2^ = 0.07 in the Lusitano [28]. An estimation of h^2^ for the upper line length was reported for the Old Kladruber horse [31] and was higher (h^2^ = 0.28) than in IHDH.

A final morphologic overall score was also considered in other horse breeds to summarize the conformation evaluation. The type in the Noriker draught horse showed a h^2^ = 0.37 close to the IHDH overall score. Lower estimates were found in the Lusitano horse ([28]; h^2^ = 0.14) and in the Sardinian Anglo Arab Horse ([36]; h^2^ = 0.23). Different aspects are likely to be valued for providing an overall morphologic judgement in breeds with different purposes and the heritabilities estimated, as well as the genetic correlations with the other traits scored, reflect the selection purposes of the breeds [2].

The overall score showed in IHDH a positive genetic correlation only with the traits related to muscular development and this indicates the importance of such traits in the final appreciation of candidate mares and stallions. Similar high genetic correlations between the overall score and the other traits of interest were also found in the Noriker horse [6].

The genetic correlations among the linear type traits scored in IHDH reflect on some extent the breeding purposes of the breed. The high and negative genetic correlation between head and bone incidence is consistent with the fact that in IHDH selection they are preferred horses with not much voluminous head. This is important because bone incidence directly correlates with the yield at abattoir, lower if bone incidence in carcass is greater. High and positive genetic correlations between head and temperament/movement, underlining a general good appearance of the animals in aspect and movement, is desirable in IHDH selection [9]. Similarly, in the Noriker a genetic correlation of 0.66 between head and movement was found [6]. The head size is positive correlated with temperament/movement because neck and head are important for the balance of the horse and subsequently they influence the movement. In IHDH selection, horses with a not too voluminous head and not too short neck, but with good development of muscular mass, are preferred.

Genetic correlations between head and traits related to muscularity excluding fore diameter did not differ from zero. Similarly, in the Lusitano [28] low genetic correlations between head–neck and chest-thorax (*r_g_* = 0.081) were found. However, the genetic correlation between head–neck and croup was moderate (*r_g_* = 0.24). In the Andalusian [14], genetic correlations of *r_g_* = 0.22 for head–neck with both chest-thorax and croup-tail were found. In the Noriker [14], a genetic correlation of 0.74 was found between the morphologic measures of head and chest circumference and of 0.58 between head and rear quarter. A correlation of *r_g_* = 0.52 between the morphometric measures of head length and chest width was found in Iranian Thoroughbred horses [25]. Differences in correlations may be due to the evaluation system (morphometric measurements or linear scoring) and to the different ways to score the head in different breeds: in IHDH the highest scores are provided to animals with a smaller head, whereas in other breeds different aspects as the shape of the head are valued [28].

The high and positive genetic correlations of frame size with thorax depth and rear and fore diameter arise because the evaluation of frame size also include the two traits. Conversely, the high and positive genetic correlations of fleshiness with fore and rear diameter and thorax depth reflect the fact that wide diameter offer more space for muscle masses and the thorax develops consistently with diameter. Fleshiness had a low and not different from zero genetic correlation with bone incidence, as expected since bone incidence is measured in relation to the muscle development.

Briefly looking at horse literature, an high value of *r_g_* (0.52) between chest-thorax and was found in Lusitano [28] and a value close to one was found between chest and thorax in the Pantaneiro horse [33]. An average genetic correlation between chest width and croup width of *r_g_* = 0.37 was reported in the Banei Draught Racehorse [15].

The genetic correlations among traits related to conformation correctness suggest strong relationships between the defects occurred in different body parts, between the upper line direction and in the rear feet and between fore and rear feet. An example of high genetic correlation (*r_g_* = 0.61) between correctness and hind feet was reported in the Noriker horse [6].

The genetic trends of traits are consistent with traits heritability, the genetic correlations among traits, the selection purposes of the breed and the biologic meaning of the linear scoring. Linear type traits are typically defined with the extreme scoring corresponding to biologic extremes, therefore a minimum score of one measured today is different from the score of one measured years ago, in terms, e.g., of body measurement of the trait. This is due to the occurrence of genetic improvement, that is able to change the average value of traits over time [51]. The trend is constantly positive in traits included within the selection index, as well as in traits highly genetically correlated with them. In IHDH, breeders prefer horses that are very reactive to environmental stimuli and exhibiting a regular trout exhibited during the evaluation. These characteristics correspond to high scores for temperament/movement (Supplementary Table S1). The trait showed indeed a positive increase over time. This increment is lower than in other traits like fleshiness due to the lower heritability. Bone incidence, negatively correlated with head size, showed a trend close to zero. The slight genetic variation in correctness traits is because traits have intermediate optima and the best individual breeding values for these traits are the mean ones. The positive increase of traits with intermediate optima as upper line length and direction is thus not desirable, suggesting a possible inclusion of correctness within the TMI in the medium-long term. The genetic variations over the years observed in this study follow the positive genetic trends already provided for traits scored at six months of age [9] and suggest that the selection carried out in young foals is effective also for the genetic improvement for traits scored at 30 months of age. Traits showing the greater increase are, as a matter of fact, the ones included in the TMI built on young foals’ traits and with high heritabilities, like fore diameter, rear diameter and fleshiness. The overall score—subjectively valued only at 30 months as a summarization of all the important characteristics of the breed—is also showing a positive increase. A genetic evaluation based on traits scored at the age of young mares and foals (currently just scored and not used for genetic improvement) may therefore be factual as well as the current one carried out on young foals. Evaluations at 30 months are generally done on nearly half of animals that are available for the evaluations in foals, but the number is still adequate for robust genetic analysis. Preliminary analyses of genetic correlations between linear type traits scored at six vs. 30 months have shown an overall genetic correlation of 0.80 between the same trait scored at the different ages (data not shown). This correlation is higher for body size and conformation characteristics like head size, frame size, upper line length and upper line direction (average *r_g_* = 0.88), lower for meat traits like fleshiness and, indirectly, thorax depth (average *r_g_* = 0.61) and intermediate for temperament. The evaluations at the two ages are able to take into account different aspects of animal career. The six-month evaluation intends to appreciate the meat attitude of the breed. Fleshiness is more heritable indeed in young animals, as well as traits related to the skeletal development, including fore diameter and rear diameter. On the other hand, also the heavy draught is important and evaluating the traits more related to this attitude (head size and temperament/movement) at 6 months or 30 months does not provide many differences, due to the similar heritabilities of traits [9] and to the high correlations shown in preliminary analysis. Finally, evaluation at 30 months rewards animals with a good conformation to generate foals. A proper knowledge of genetic correlation among traits at six and 30 months, currently under study, will be useful for the future to substitute the evaluation at six months with 30 months. That will allow to reduce evaluated animals and consistently the costs. The diminishing in the national funding for breeders organizations and breeders that was observed in the last years for all livestock species is producing indeed its effects in horse breeds’ management and effective strategies able to reduce the costs, but maintaining the quality of the management decisions are going to be essential for the close future.

## 5. Conclusions

To conclude, it is possible to observe that genetic parameters of traits recorded at 30 months of age are consistent with estimates obtained in traits recorded in young foals [9] and currently used for genetic improvement. Genetic evaluation in IHDH is based on a linear type evaluation of 11 traits scored at six months of age, five of them included in the selection index of the breed. At 30 months age the evaluation is performed again on the same traits and three further traits of legs correctness, as well as an overall score for morphology.

The results about morphologic evaluation indicate that the goal of selection is to obtain an animal with a good muscular mass, but elegant and brilliant in the movements. The traits included in the TMI are head size, temperament/movement, fleshiness, fore diameter, rear diameter that have a medium high heritability (ranging from h^2^ = 0.21 to h^2^ = 0.31). The traits involved in the muscular development that are fleshiness and fore and rear diameter, had moderate high heritability estimates, indicating that selection can be used for these traits and a suitable response will be found. These traits are all positively correlated, and the genetics correlations were very high, ranging from *r_g_* = 0.74 to *r_g_* = 0.91, while phenotypic correlations were lower, ranging from *r_g_* = 0.47 to *r_g_* = 0.51. Most of the genetic correlations between traits included in the selection index are moderate-high, meaning that selection for one of these traits should result in an increase in the other traits of interest. Positive genetic trends were observed for traits of selection interest despite that breeding values are estimated on traits scored at young age. Evaluations at the age of 30 months are however mandatory for the final admission at stud book of candidate stallions and mares. This study has shown that the use of traits scored in young mares and stallions instead that in foals is feasible in IHDH selection.

## Figures and Tables

**Table 1:** Overview of morphologic evaluation in some horse breeds.

Breed	Purpose	Evaluation ^1^	Age	Ref.
Banei	Draught	BM	≥2 years	[15]
Bardigiano horse	Draught/Sport	LS/SJ	≥3 years	[16,17]
Belgian Warmblood horse	Sport	LS	3–4 years	[18]
Campolina horse	Sport	BM	22–52 months	[19]
Czech Warmblood	Sport	LS	3–4 years	[20]
Czech-Moravian Belgian	Draught	LS/BM	≥3 years	[12,21]
Dutch Warmblood horse	Sport	LS; SJ	3–7 years	[4,11]
Franches-Montagnes horse	Sport	DI	3 years	[22]
Haflinger	Draught/Sport	LS	30 months	[5]
Hanoverian Warmblood horse	Sport	SJ/BM	≥3 years	[23]
Icelandic horse	Sport/Leisure	BM/DI	4–18 years	[24]
Iranian Thoroughbred horse	Sport	BM	2–4 years	[25]
Italian Heavy Draught Horse	Draught/Meat	LS	6 months	[9,10]
Lipizzan horse	Sport	BM; LS/DI	≥4 years	[26,27]
Lusitano horse	Sport	SJ/BM	≥3 years	[28]
Menorca horse	Sport	BM/DI	≥3 years	[29]
Murgese horse	Draught	BM	30 months	[30]
Noriker	Draught	BM; LS	≥3 years	[6,12]
Old Kladruber	Sport	SJ/BM	≥4 years	[31,32]
Pantaneiro horse	Draught/Meat	LS		[33]
Posavje	Draught	LS/BM	30–60 months	[13]
Pura Raza Español horse (Andalusian)	Sport	BM; LS	≥3 years	[14,34,35]
Sardinian Anglo Arab horse	Sport	BM		[36]
Shetland pony	Sport/Leisure	LS	3 years	[37]
Silesian Noriker	Draught	LS	≥3 years	[12]
Spanish Arab horse	Sport	DI	≥3 years	[38]
Spanish heavy horse breeds ^2^	Draught/Meat	BM	≥4 years	[39]
Swedish Warmblood horse	Sport	LS	3–4 years	[40,41]
Wielkopolski	Sport	BM		[42]

^1^ BM = body measurements; LS = linear scale; SJ = subjective judgement; DI = digital images. ^2^ Hispano-Breton; Jaca Navarra; Burguete; Cavall Pirinenc Català.

**Table 2:** Descriptive statistics of the 15 linear traits scored in 6691 the Italian Heavy Draught Horse (IHDH) horses.

Trait	Mean ± SD	Skewness	Kurtosis	Minimum	Maximum
Head size (HS)	3.04 ± 0.64	0.05	−0.14	Heavy	Light
Temperament/movement (Te/m)	3.29 ± 0.54	0.17	0.45	Lymphatic	Nevrile
Frame size (FS)	3.20 ± 0.71	0.07	−0.25	Little	Large
Fleshiness (Fl)	3.28 ± 0.54	−0.03	0.09	Poor	Excellent
Bone incidence (BI)	2.88 ± 0.39	−0.76	2.53	Fine-boned	Heavy-boned
Thorax depth (TD)	3.55 ± 0.53	−0.19	−0.38	Little	Large
Fore diameter (FD)	2.93 ± 0.65	0.17	−0.25	Narrow	Wide
Rear diameter (RD)	3.37 ± 0.56	0.01	−0.28	Narrow	Wide
Upper line length (UL)	3.28 ± 0.45	0.65	−0.37	Short	Long
Upper line direction (UD)	2.85 ± 0.36	−1.80	1.97	Kyphotic	Curved
Legs side view (LS)	2.58 ± 0.47	−0.28	−1.06	Sickle	Straight
Fore feet (FF)	3.22 ± 0.51	0.32	0.52	Diverging	Converging
Rear feet (RF)	3.01 ± 0.42	0.03	2.88	Diverging	Converging
Hind legs back view (HL)	2.90 ± 0.33	−1.73	4.40	Diverging	Converging
Overall score (OS)	2.05 ± 0.79	0.28	−0.51	Fair	Excellent

**Table 3:** Genetic (σ_a_^2^), residual (σ_e_^2^), phenotypic variance (σ_p_^2^), heritability (h^2^) and their standard errors (SE) for traits under study.

Trait	σ_a_^2^ (SE)	σ_e_^2^ (SE)	σ_p_^2^ (SE)	h^2^ (SE)
Head size (HS)	10.09 (1.15)	22.20 (0.92)	32.29 (0.72)	0.31 (0.032)
Temperament/movement (Te/m)	4.75 (0.71)	18.01 (0.64)	22.77 (0.49)	0.21 (0.029)
Frame size (FS)	13.98 (1.35)	20.92 (1.00)	34.9 (0.81)	0.40 (0.033)
Fleshiness (Fl)	5.47 (0.71)	16.21 (0.61)	21.68 (0.47)	0.25 (0.030)
Bone incidence (BI)	1.68 (0.37)	11.31 (0.37)	12.99 (0.27	0.13 (0.028)
Thorax depth (TD)	4.31 (0.64)	16.00 (0.58)	20.31 (0.43)	0.21 (0.030)
Fore diameter (FD)	8.95 (1.00)	19.57 (0.81)	28.52 (0.63)	0.31 (0.031)
Rear diameter (RD)	6.26 (0.79)	17.16 (0.67)	23.42 (0.51)	0.27 (0.031)
Upper line length (UL)	1.58 (0.40)	14.79 (0.44)	16.37 (0.34)	0.10 (0.024)
Upper line direction (UD)	0.28 (0.17)	8.41 (0.22)	8.69 (0.17)	0.03 (0.019)
Legs side view (LS)	1.94 (0.43)	13.81 (0.44)	15.75 (0.33)	0.12 (0.026)
Fore feet (FF)	1.85 (0.53)	21.3 (0.61)	23.15 (0.47)	0.08 (0.023)
Rear feet (RF)	0.40 (0.24)	13.98 (0.35)	14.37 (0.29)	0.03 (0.017)
Hind legs back view (HL)	0.54 (0.19)	7.98 (0.22)	8.53 (0.17)	0.06 (0.022)
Overall score (OS)	13.47 (1.47)	30.22 (1.20)	43.69 (0.96)	0.31 (0.030)

**Table 4:** Estimates of genetic correlations (above diagonal) and phenotypic correlations (below diagonal), between each trait pairs considered in the study. Significant correlations are bolded. Standard errors of traits are reported in an extended version of table (Supplementary Table S3).

Trait1	HS	Te	FS	F	BI	TD	FD	RD	UL	UD	LS	FF	RF	HL	OS
HS		**0.67**	0.13	0.12	**−0.64**	0.14	**0.18**	0.1	−0.01	−0.1	0.12	−0.15	−0.23	−0.21	**0.45**
Te	**0.3**		**0.24**	0.09	**−0.74**	0.15	0.13	**0.09**	0.18	0.05	**0.25**	−0.19	−0.15	−0.12	**0.47**
FS	**0.08**	**0.13**		**0.45**	**0.22**	**0.71**	**0.52**	**0.73**	**0.41**	**0.38**	−0.03	0.13	0.03	−0.1	**0.85**
Fl	**0.11**	**0.09**	**0.31**		0.07	**0.55**	**0.74**	**0.91**	0.09	−0.18	**−0.33**	**0.33**	0.09	0.04	**0.61**
BI	**−0.29**	**−0.18**	**0.05**	**−0.08**		0.13	0.03	0.15	0.01	0.22	−0.23	**0.36**	0.4	0.13	−0.13
TD	**0.09**	**0.05**	**0.39**	**0.3**	0.01		**0.56**	**0.74**	0.01	−0.09	**−0.29**	0.14	0.08	0.13	**0.72**
FD	**0.14**	**0.11**	**0.36**	**0.47**	**−0.04**	**0.36**		**0.76**	0.23	0.05	**−0.27**	**0.35**	0.26	0.18	**0.7**
RD	**0**	**0.07**	**0.44**	**0.51**	−0.01	**0.4**	**0.5**		**0.34**	−0.01	**−0.25**	0.16	0.1	−0.25	**0.77**
UL	**−0.04**	**0.03**	**0.13**	**−0.05**	**0.05**	−0.03	**0.04**	**0.03**		0.14	0.04	0.15	−0.17	0.16	0.19
UD	0.01	**0.03**	**0.06**	−0.03	**0.05**	−0.02	**−0.04**	−0.01	**−0.06**		0.1	−0.12	**−0.99**	−0.21	0.23
LS	**0.1**	**0.11**	−0.03	**−0.04**	**−0.1**	**−0.04**	−0.01	**−0.06**	**−0.07**	0.02		−0.13	−0.24	**0.39**	0.05
FF	−0.01	−0.03	**0.04**	**0.08**	0.01	**0.06**	**0.12**	**0.11**	0.02	−0.01	−0.03		**0.56**	−0.06	0.1
RF	−0.02	−0.01	0.01	0.02	0.02	0.02	0.03	0.01	0.01	0.01	−0.02	**0.09**		0.49	−0.11
HL	**0.03**	0.01	−0.01	0.02	0.01	0.01	0.04	−0.01	−0.01	−0.01	**0.08**	0.02	**0.1**		−0.04
OS	**0.31**	**0.3**	**0.58**	**0.46**	**−0.09**	**0.41**	**0.52**	**0.54**	0.01	**0.04**	**0.06**	**0.06**	0.01	**0.04**	

^1^ HS = Head size; Te/M = Temperament/movement; FS = Frame size; Fl = Fleshiness; BI = Bone incidence; TD = Thorax depth; FD = Fore diameter; RD = Rear diameter; UL = Upper line length; UD = Upper line direction; LS = Legs side view; FF = Fore feet; RF = Rear feet; HL = Hind legs back view; OS = Overall score.

## Supplementary Materials

The following are available online at https://www.mdpi.com/2076-2615/10/6/1099/s1, Table S1: Description of the linear type traits evaluated on the Italian Heavy Draught Horse population, Table S2: Results of preliminary ANOVA performed on the 15 linear traits considered in the analyses, Table S3: Estimates of genetic correlations (above diagonal) and phenotypic correlations (below diagonal), with relative standard errors (in brackets), between each trait pairs considered in the study, Figure S1: Mare and foal of the Italian Heavy Draught Horse.
