# Supplementary material for: Genetic Parameters of Linear Type Traits Scored at 30 Months in Italian Heavy Draught Horse
Source: Animals (Basel). 2020 Jun 25;10(6):1099. doi: 10.3390/ani10061099 (PMC7341255; doi:10.3390/ani10061099)
Supplement: Supplementary file 1 [file animals-10-01099-s001.pdf]

Supplementary material for manuscript:

Genetic parameters of linear type traits scored at 30 months in Italian Heavy Draught Horse

Fabio Folla, Cristina Sartori*, Enrico Mancin, Giuseppe Pigozzi and Roberto Mantovani

* Correspondence: cristina.sartori@unipd.it

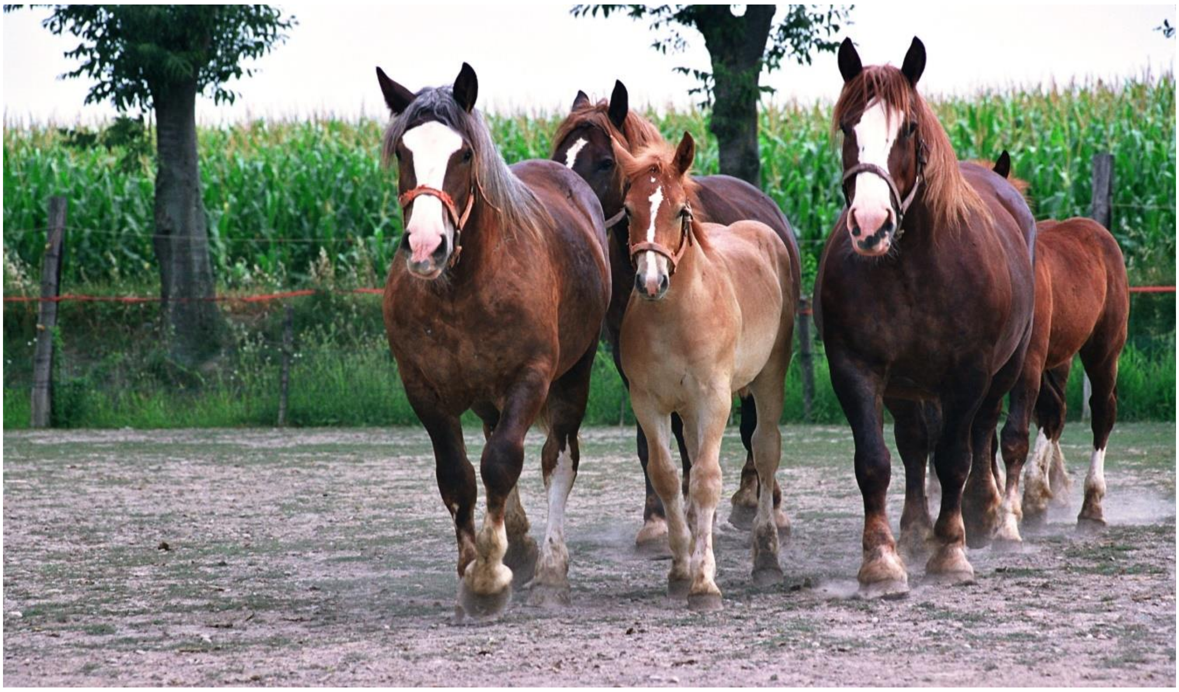

Supplementary Figure 1. Mares and foals of Italian Heavy Draught Horse (courtesy of Cinzia Stoppa)

Supplementary Table 1. Description of the linear type traits evaluated on the Italian Heavy Draught Horse population.

Trait	Evaluation	Descriptor Minimum (1)	Descriptor Maximum (5)
Head size	Evaluate Head and Neck. Head should be not too voluminous and neck should contain a good muscular mass.	Light	Heavy
Temperament/Movement	Evaluate the reactivity to stimuli and the movements. Animals are preferred very reactive to environmental stimuli. Lymphatic elements should be underlined and avoided.	Lymphatic	Nevrile
Frame size	Evaluate the rate between trunk development and height. The minimum height at withers should be overcome, such as transverse diameters and thorax depth is the target for a well-shaped animal.	Little	Large
Fleshiness	Evaluate muscular mass at shoulder, back, rump, crouch, and thigh. Subjects are required to present a good muscular mass.	Poor	Excellent
Bone incidence	Evaluate the incidence of the head and the distal parts of the limbs on the whole body. Coarse subjects should be underlined and avoided.	Fine boned	Heavy boned
Thorax depth	Evaluate the height of thorax, i.e., measure the height between withers and sternum.	Little	Large
Fore diameters	Evaluate the width of chest by looking at the width at level of the scapular-humeral articulation.	Narrow	Wide
Rear diameters	Evaluate the width of croup (i.e., at ilium and ischium).	Narrow	Wide
Upper line length	Evaluate the length of back and loins. Short animals are preferred as respect to longer animals.	Short	Long
Upper line direction	Evaluate the direction of the upper line. Kyphosis or lordosis are defects that should be underlined and avoided.	Kyphotic	Inward spine
Rear legs side view	Evaluate the hock articulation. Subjects with sickle or straight defect should be underlined and avoided.	Sickle	Straight
Fore feet [1]	Evaluate the tethers and hooves of the forelegs. Divergent or convergent feet should be underlined and avoided.	Diverging	Converging
Rear feet [1]	Evaluate the tethers and hooves of the rear legs. Divergent or convergent feet should be underlined and avoided.	Diverging	Converging
Hind legs back view [1]	Evaluate the hocks from the back view. Diverging or converging hooks should be underlined and avoided	Converging	Diverging

[1] Traits evaluated only in 30-months animals

Supplementary Table 2. Results of preliminary ANOVA performed on the 15 linear traits considered in the analyses. Sum of squares are reported for each effect included in the model. Residual variance (root mean square error) for each trait is also reported.

**Trait**	**Effect**			**Residual variance**
	**HYC**	**SEX**	**AGE**	
Head size (HS)	0.649***	6.063***	0.502	0.32
Temperament/Movement (Te/M)	0.493***	0.243	0.864**	0.22
Frame size (FS)	0.893***	29.52***	1.645***	0.34
Fleshiness (Fl)	0.486***	9.279***	1.571***	0.21
Bone incidence (BI)	0.232***	2.088***	0.032	0.13
Thorax depht (TD)	0.513***	0.077	0.841**	0.20
Fore diameters (FD)	0.758***	25.76***	2.255***	0.28
Rear diameters (RD)	0.534***	<0.001	2.204***	0.23
Upper line lenght (UL)	0.313***	1.939***	0.135	0.16
Upper line direction (UD)	0.259***	2.424***	0.008	0.09
Legs side view (LS)	0.403***	0.190	0.379*	0.15
Fore feet (FF)	0.341***	3.798***	0.451	0.23
Rear feet (RF)	0.261***	2.321***	0.108	0.14
Hind legs back view (HL)	0.169***	0.002	0.104	0.08
Overall score (OS)	1.142***	0.277	5.774***	0.43

[1] HYC is the herd-year-classifier effect (1663 levels); SEX is the sex effect (male or female), AGE is the class of age at the time of linear evaluation (5 levels); *$P < 0.05$; **$P < 0.01$; ***$P < 0.001$; when absent not significant.

Supplementary Table 3. Estimates of genetic correlations (above diagonal) and phenotypic correlations (below diagonal), with relative standard errors (in brackets), between each trait pairs considered in the study.

Trait[1]	HS	Te	FS	F	BI	TD	FD	RD	UL	UD	LS	FF	RF	LB	OS
HS		**0.67 (0.069)**	0.13 (0.074)	0.12 (0.085)	**-0.64 (0.088)**	0.14 (0.092)	**0.18 (0.078)**	0.10 (0.085)	-0.01 (0.124)	-0.10 (0.185)	0.12 (0.111)	-0.15 (0.130)	-0.23 (0.213)	-0.21 (0.142)	**0.45 (0.069)**
Te	**0.30 (0.014)**		**0.24 (0.083)**	0.09 (0.098)	**-0.74 (0.104)**	0.15 (0.104)	0.13 (0.090)	**0.09 (0.097)**	0.18 (0.140)	0.05 (0.219)	**0.25 (0.122)**	-0.19 (0.146)	-0.15 (0.228)	-0.12 (0.167)	**0.47 (0.076)**
FS	**0.08 (0.016)**	**0.13 (0.015)**		**0.45 (0.069)**	**0.22 (0.102)**	**0.71 (0.060)**	**0.52 (0.061)**	**0.73 (0.051)**	**0.41 (0.108)**	**0.38 (0.188)**	-0.03 (0.106)	0.13 (0.123)	0.03 (0.189)	-0.10 (0.137)	**0.85 (0.034)**
Fl	**0.11 (0.015)**	**0.09 (0.015)**	**0.31 (0.014)**		0.07 (0.119)	**0.55 (0.081)**	**0.74 (0.053)**	**0.91 (0.039)**	0.09 (0.131)	-0.18 (0.192)	**-0.33 (0.125)**	**0.33 (0.127)**	0.09 (0.209)	0.04 (0.154)	**0.61 (0.061)**
BI	**-0.29 (0.014)**	**-0.18 (0.014)**	**0.05 (0.015)**	**-0.08 (0.015)**		0.13 (0.126)	0.03 (0.110)	0.15 (0.115)	0.01 (0.168)	0.22 (0.250)	-0.23 (0.149)	**0.36 (0.170)**	0.40 (0.275)	0.13 (0.199)	-0.13 (0.108)
TD	**0.09 (0.015)**	**0.05 (0.015)**	**0.39 (0.013)**	**0.30 (0.014)**	0.01 (0.015)		**0.56 (0.072)**	**0.74 (0.061)**	0.01 (0.140)	-0.09 (0.212)	**-0.29 (0.131)**	0.14 (0.146)	0.08 (0.221)	0.13 (0.164)	**0.72 (0.060)**
FD	**0.14 (0.015)**	**0.11 (0.015)**	**0.36 (0.014)**	**0.47 (0.012)**	**-0.04 (0.015)**	**0.36 (0.013)**		**0.76 (0.049)**	0.23 (0.123)	0.05 (0.195)	**-0.27 (0.116)**	**0.35 (0.122)**	0.26 (0.193)	0.18 (0.145)	**0.70 (0.049)**
RD	**0.0 (0.015)**	**0.07 (0.015)**	**0.44 (0.012)**	**0.51 (0.011)**	-0.01 (0.015)	**0.40 (0.013)**	**0.50 (0.011)**		**0.34 (0.126)**	-0.01 (0.201)	**-0.25 (0.118)**	0.16 (0.134)	0.10 (0.207)	-0.25 (0.149)	**0.77 (0.047)**
UL	**-0.04 (0.015)**	**0.03 (0.015)**	**0.13 (0.015)**	**-0.05 (0.015)**	**0.05 (0.015)**	-0.03 (0.015)	**0.04 (0.015)**	**0.03 (0.015)**		0.14 (0.273)	0.04 (0.169)	0.15 (0.193)	-0.17 (0.265)	0.16 (0.209)	0.19 (0.122)
UD	0.01 (0.015)	**0.03 (0.014)**	**0.06 (0.015)**	-0.03 (0.015)	**0.05 (0.014)**	-0.02 (0.014)	**-0.04 (0.015)**	-0.01 (0.015)	**-0.06 (0.014)**		0.10 (0.253)	-0.12 (0.285)	**-0.99 (0.007)**	-0.21 (0.327)	0.23 (0.201)
LS	**0.10 (0.015)**	**0.11 (0.015)**	-0.03 (0.015)	**-0.04 (0.015)**	**-0.10 (0.015)**	**-0.04 (0.015)**	-0.01 (0.015)	**-0.06 (0.015)**	**-0.07 (0.014)**	0.02 (0.014)		-0.13 (0.175)	-0.24 (0.259)	**0.39 (0.182)**	0.05 (0.110)
FF	-0.01 (0.015)	-0.03 (0.015)	**0.04 (0.015)**	**0.08 (0.015)**	0.01 (0.015)	**0.06 (0.015)**	**0.12 (0.015)**	**0.11 (0.015)**	0.02 (0.014)	-0.01 (0.014)	-0.03 (0.014)		**0.56 (0.282)**	-0.06 (0.225)	0.10 (0.127)
RF	-0.02 (0.015)	-0.01 (0.014)	0.01 (0.015)	0.02 (0.014)	0.02 (0.014)	0.02 (0.014)	0.03 (0.015)	0.01 (0.014)	0.01 (0.014)	0.01 (0.012)	-0.02 (0.014)	**0.09 (0.014)**		0.49 (0.309)	-0.11 (0.203)
LB	**0.03 (0.015)**	0.01 (0.015)	-0.01 (0.015)	0.02 (0.015)	0.01 (0.014)	0.01 (0.015)	0.04 (0.015)	-0.01 (0.015)	-0.01 (0.014)	-0.01 (0.014)	**0.08 (0.014)**	0.02 (0.014)	**0.10 (0.014)**		-0.04 (0.143)
OS	**0.31 (0.014)**	**0.30 (0.014)**	**0.58 (0.010)**	**0.46 (0.012)**	**-0.09 (0.015)**	**0.41 (0.013)**	**0.52 (0.011)**	**0.54 (0.011)**	0.01 (0.015)	**0.04 (0.014)**	**0.06 (0.015)**	**0.06 (0.015)**	0.01 (0.014)	**0.04 (0.015)**	

[1] HS = Head size; Te/M = Temperament/Movement; FS = Frame size; Fl = Fleshiness; BI = Bone incidence; TD = Thorax depht; FD = Fore diameters; RD = Rear diameters; UL = Upper line lenght; UD = Upper line direction; LS = Legs side view; FF = Fore feet; RF = Rear feet; HL = Hind legs back view; OS = Overall score.
